# Long noncoding RNA LINC01811 sponges miR-214-3p and upregulates YAP1 thereby promoting the migration and invasion of colorectal cancer

**DOI:** 10.1007/s13205-025-04292-8

**Published:** 2025-04-10

**Authors:** Li Zhu, Wen Shi, Yiminjiang Tuoheti, Guo-jie Gong, Min Chen, Zong-hua Liang, Abuduweili Abudureheman, Wei-ge Gao

**Affiliations:** Department of Colorectal Surgery Ward, People’s Hospital of Xinjiang Uygur Autonomous Region, No. 91 Tianchi Road, Urumqi, 830000 China

**Keywords:** Colorectal cancer, LncRNA LINC01811, MiR-214-3p, *YAP1*, Progression

## Abstract

Long non-coding RNAs (lncRNAs) exert significant influence on the development of cancer. However, their role in colorectal cancer (CRC) is not fully clarified. The expression levels of LINC01811 in CRC samples were determined using differential expression analysis and validated by RT-qPCR assays. Transwell assays were conducted to investigate the biological function of LINC01811 in CRC. To elucidate the mechanism by which LINC01811 acts as a molecular sponge for miR-214-3p and regulates *YAP1* expression, binding site analysis, Luciferase reporter assay, RT-qPCR, and Western blotting were employed. We identified a novel oncogenic lncRNA LINC01811 in CRC tissues and cell lines. Our results showed that the suppression of LINC01811 significantly reduced CRC cell invasion and migration by regulating epithelial-mesenchymal transition-related markers, including MMP2, MMP9, vimentin, and E-cadherin in vitro. Furthermore, LINCO1811 modulated *YAP1* expression by sequestering miR-214-3p, thereby promoting CRC progression by suppressing its activity. In summary, this study identified a novel lncRNA LINC01811 involved in CRC progression through the miR-214-3p/*YAP1* axis.

**Supplementary Information:**

The online version contains supplementary material available at 10.1007/s13205-025-04292-8.

## Introduction

Colorectal cancer (CRC) is a prevalent solid tumor in the digestive system, often found at the junction of the rectum and sigmoid colon, and is characterized by strong aggressiveness and high malignancy (Dekker et al. 2019). In 2024, approximately 152,854 new cases of CRC and 53,010 CRC-related deaths are projected to occur in the United States (Siegel et al. 2024). The incidence of bowel cancer in both men and women has fallen in recent years as colonoscopy has become more widely available (Siegel et al. 2021). Notably, the global decline in CRC incidence masks an increase in the incidence among adults under 65 years of age (Thrift and Gudenkauf 2020). The incidence of colorectal cancer among young adults (under 55 years old) increases by 1–2% each year (Siegel et al. 2024). In the late 1990s, CRC was the fourth leading cause of cancer death in men and women under 50; it is now the leading cause of cancer death in men and the second cause of cancer death in women (Siegel et al. 2024). Treatment methods such as surgical resection, adjuvant chemotherapy, and immunotherapy have significantly improved the effectiveness of CRC treatment (Johdi and Sukor 2020; Biller and Schrag 2021). However, current approaches are still insufficient in improving clinical outcomes and prognosis for CRC patients (Siegel et al. 2017). This underscores the importance of developing and implementing new therapeutic strategies.

Long non-coding RNA (lncRNA) refers to RNA molecules that are more than 200 nucleotides long and have been shown to play a crucial role in regulating various cellular processes by controlling gene expression at the transcription and translation levels (Choi et al. 2019; Lozano-Vidal et al. 2019). Studies have demonstrated that lncRNA is often dysregulated in the metastasis and progression of CRC and holds promise as a prognostic marker and therapeutic target for tumors (Ni et al. 2019a, b; Wang et al. 2019, 2022). More specifically, lncRNA can modulate the translation, stability, and degradation of messenger RNA (mRNA) and serve as competitive endogenous RNA (ceRNA) by binding to microRNAs (miRNAs) to regulate gene expression (Yan and Bu 2021). For instance, certain lncRNAs like UCA1 and RP11-757G1.5 have been shown to competitively bind to specific miRNAs, preventing them from interacting with their target mRNAs and leading to upregulation of downstream genes that promote CRC cell proliferation and metastasis (Bian et al. 2016; Zhu et al. 2020). Additionally, lncRNA UICLM has been found to upregulate the expression of zinc finger E-box binding homeobox 2 (ZEB2) protein by binding to mir-215, thereby enhancing cell proliferation, invasion, and liver metastasis in CRC (Chen et al. 2017). Despite these findings, the molecular mechanisms of lncRNAs remain complex, and there are still many unknown regulatory relationships that require further investigation. Therefore, a more comprehensive study on the role of lncRNA in CRC is necessary to fully understand its impact and potential therapeutic implications.

In this study, we have identified a novel lncRNA, LINC01811, in CRC tissues and cell lines, which demonstrates elevated expression levels in CRC tissues. The mechanisms underlying the role of LINC01811 in CRC was explored. Furthermore, the impact of the regulatory axis involving LINC01811, miR-214-3p, and *YAP1* on the progression of CRC was analyzed. These findings illuminate potential therapeutic opportunities presented by the novel LINC01811/miR-214-3p/*YAP1* axis in CRC progression.

## Methods

### Data source

RNA sequencing (RNA-Seq) data from 456 CRC cases were extracted from The Cancer Genome Atlas (TCGA, https://tcga-data.nci.nih.gov/tcga/) database, including 456 CRC tissues and 41 para-cancerous tissues. The gene transfer format (GTF) annotation file for long non-coding RNAs (lncRNAs) (gencode.v34.long_noncoding_RNAs.gtf.gz) was collected from the GENECode website (https://www.gencodegenes.org/). lncRNA expression data was extracted from RNA-seq data according to the correspondence between lncRNA and Ensemble ID in the annotation file.

### Differential expression analysis

Differential expression analysis between CRC and adjacent tissues was conducted using the edgeR package in R language, following the removal of lncRNAs with zero expression in over 50% of the samples (Robinson et al. 2010). The differentially expressed lncRNAs (DElncRNAs) between the two groups were screened using |Log_2_FC|> 3 and FDR < 0.05.

### Prediction of lncRNA target genes and functional enrichment analysis

The starBase database (version 3.0, http://starbase.sysu.edu.cn/) (Li et al. 2014) and the lncRNA2target database (version 2.0, http://123.59.132.21/lncRNA2target/) (Cheng et al. 2019) were utilized to predict the target genes of lncRNAs. Enrichment analysis for the target genes involved utilizing the enrichGO function in the “clusterProfiler” package of R language to examine Gene Ontology (GO) categories. Additionally, the enrichKEGG function was used to conduct Kyoto Encyclopedia of Genes and Genomes (KEGG) enrichment analysis. Identification of significantly enriched GO terms and KEGG pathways was based on an adjusted P value < 0.05.

### Cancer tissues and adjacent tissues from clinical patients

Fifteen pairs of tumors and adjacent tissues from CRC patients were obtained from the People’s Hospital of Xinjiang Uygur Autonomous Region. Each patient agreed to participate and signed the informed consent form. All studies were endorsed by the Ethical Committee of People’s Hospital of Xinjiang Uygur Autonomous Region (no. YBK2022090213), adhered to the Helsinki guidelines, and obtained informed consent from all participants. The clinical information of the patients is shown in Table S1.

### Cell culture and transfection

Four CRC cell lines (HT29, HCT15, LOVO, Caco-2) and a normal human colonic epithelial cell line NCM460 were acquired from the American Type Culture Collection (ATCC) and used for experiments. The cells were cultured in DMEM medium supplemented with 10% FBS (Gibco, 25,200-072), penicillin and streptomycin mixture (Gibco, MP 1670249-M), 0.25% Trypsin (Gibco, 25,200-072) at 37 °C with 5% CO_2_. Cell lines were maintained under standard conditions to ensure their viability and integrity for the duration of the study.

Small interference RNA (siRNA) targeting LINC01811 (si-LINC01811#1, si- LINC01811#2, si-LINC01811#3), si-NC, miR-214-3p inhibitor, miR-214-3p inhibitor-NC, miR-214-3p mimic, and miR-214-3p mimic-NC were obtained from Ribobio (Guangzhou, China). The siRNAs and miRNA mimics and inhibitors were successfully transfected into HT29 cells utilizing Lipo8000™ Transfection Reagent (Beyotime, Shanghai, China), following the manufacturer’s instructions. The sequences were shown as follows:

si-LINC01811#1 F: 5′-GCUAAGUCUUCCUAUUGUACU-3′

R: 5′-UACAAUAGGAAGACUUAGCUU-3′

si-LINC01811#2 F: 5′-GAAUUGUUGUGAAACUUAAAU-3′

R: 5′-UUAAGUUUCACAACAAUUCAA-3′

si-LINC01811#3 F: 5′-GGUAUGUUCUGUAACAGAAGA-3′

R: 5′-UUCUGUUACAGAACAUACCAU-3′

Si-NC F: 5′-UUCUCCGAACGUGUCACGTT-3′

R: 5′-ACGUGACACGUUCGGAGAATT-3′

### RNA extract and RT-qPCR assay

Total RNA was extracted from tissues and cells using TRNzol Universal Reagent (DP424, Tiangen, Beijing, China) according to the manufacturer’s instructions. For tissue samples, approximately 0.1 mg of fresh tissue was placed into a pre-cooled, sterile, and enzyme-free grinding tube and homogenized rapidly. For cell samples, cells were washed three times with pre-cooled phosphate-buffered saline (PBS; P1020, Solarbio, Beijing, China). Both tissue and cell samples were then lysed by adding 1 mL of TRNzol reagent and incubating at room temperature for 10 min. The lysate was centrifuged at 12,000 rpm for 5 min at room temperature. The supernatant was carefully collected, and 200 μL of chloroform was added, followed by vigorous mixing. The mixture was allowed to stand at room temperature for 15 min and then centrifuged at 12,000 rpm for 15 min at 4 °C. The bottom sediment, which contained the total RNA, was carefully collected. The extracted RNA was reverse transcribed using the RevertAid First Strand cDNA Synthesis Kit from Thermo Fisher Scientific. Subsequently, RT-qPCR was carried out on the PCR system (Veriti from Thermo Fisher) using SYBR green. The reaction system involved an initial denaturation step at 95 °C for 30 s, followed by 40 denaturation cycles at 95 °C for 5 s, and an extension step at 60 °C for 30 s. For miRNA analysis, the miRcute plus miRNA First-strand cDNA kit from Tiangen was utilized. The RT-qPCR protocol included an initial step at 95 °C for 15 min, followed by 40 cycles at 94 °C for 20 s, and 60 °C for 34 s. The reference genes used were U6 or β-actin, and the relative expression of the target gene was calculated using the 2^−△△Ct^ method. Additionally, the tailing method was implemented for miRNA detection, with specific sequences for RT-qPCR provided in Table 1.

**Table 1:** The RT-qPCR primer sequences and related primer sequences

Primer		Sequences (5′–3′)	Size (bp)
*miR-214-3p*	F	ACAGCAGGCACAGACAGGCAGT	
*U6*	F	CTCGCTTCGGCAGCACA	94
	R	AACGCTTCACGAATTTGCGT	
*LINC01811*	F	CTGTGCTGAGTGACTTGAATGCC	199
	R	TTCTGCTGTTTCTGGTGGTCTTC	
*YAP1*	F	CTCGGCTTCAGGTCCTCTTCC	93
	R	GAGGTGGTCTTGTTCTTATGGTTTAT	
*β-actin*	F	TGGCACCCAGCACAATGAA	167
	R	GAAGCATTTGCGGTGGACG	

### Western blotting (WB) assay

Fresh tissues or cells were lysed on ice using RIPA lysis buffer (R002, Solarbio, Beijing, China) with PMSF protease inhibitor for 15 min. The lysates were centrifuged (12,000 rpm, 10 min, 4 °C) and collected supernatant (total protein). The extracted supernatant was used for protein quantification using a BCA Protein Assay Kit (P0011, Beyotime). The SDS-PAGE technique was employed to separate the protein, which was subsequently transferred onto PVDF membranes (Millipore, IPVH00010). After this step, the membranes underwent blocking in 5% nonfat dry milk for a duration of 2 h, followed by an overnight incubation at 4 °C with a primary antibody. The membranes were then subjected to washing with 1xTBST and incubated for 1 h with a secondary antibody. The primary antibodies utilized in this research were: anti-E-cadherin antibody (CST, 3195 T, 1:1000), anti-Vimentin antibody (CST, 5741 T, 1:1000), anti-MMP9 antibody (CST, 13667 T, 1:1000), anti-MMP2 antibody (Proteintech, 66,366-1-Ig, 1:1000), and anti-YAP1 antibody (Proteintech, 66,900-1-Ig, 1:1000). The secondary antibodies used were Goat anti-rabbit IgG-HRP (Bioss, bs-0295G-HRP, 1:3000) or goat anti-mouse IgG-HRP (Bioss, bs-0296G-HRP, 1:3000). An anti-GADPH antibody (Untibody, UM4002, 1:2000) served as the internal reference in this study. The visualization of protein bands was achieved through the utilization of an ECL reagent (Beyotime, P0018S) on a Tanon5200 automated chemiluminescence image analysis system (Tanon, Shanghai, China).

### Cell invasion and migration

Transwell (Costa, 3422) assay was used in this study to evaluate the migration and invasion capabilities of the cells. By adding a cell suspension to the top chamber, either coated with Matrigel (BD, 356,234) or left uncoated and medium with 20% serum to the lower chamber, the incubation was carried out for 8 h. After fixing the chamber with methanol and staining the cells with a 2% crystal violet solution (Wuhan Google Biotechnology Co., Ltd., G1014), images were captured using a fluorescence inverted microscope (OLYMPUS, German, IX51).

### Dual-luciferase reporter assay

The LINC01811 or YAP1 3ʹ‐UTR sequences with either the miR-214-3p binding sites or mutant binding sites were incorporated into the pmirGLO reporter vector (Youbio, Changsha, China) to produce wild-type (LINC01811-WT and YAP1-WT) or mutant-type (LINC01811-MUT and YAP1-MUT) reporter vectors. These vectors were then co-transfected into HT29 cells along with miR-214-3p mimics or miR-214-3p mimic NC. The luciferase activity within the cells was measured using the dual‐luciferase reporter system as per the detection instructions.

## Results

### LINC01811 was highly expressed in CRC

**Fig. 1:**
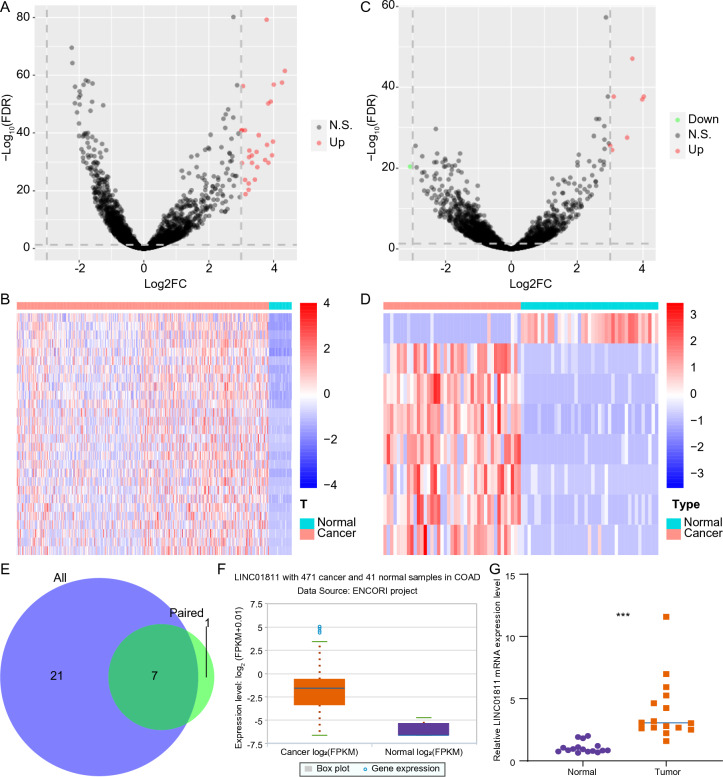
LINC01811 was highly expressed in colorectal cancer (CRC). **A** Volcano plot of differentially expressed genes (DEGs) between 456 CRC samples and 41 para-cancerous samples in the TCGA cohort. **B** Heat map of DEGs between 456 CRC samples and 41 para-cancerous samples in the TCGA cohort. **C** Volcano plot of DEGs between 41 CRC samples and 41 para-cancerous samples in the TCGA cohort. **D** Heat map of DEGs between 41 CRC samples and 41 para-cancerous samples in the TCGA cohort. **E** The result of cross-analysis between two DEG sets. **F** The expression of LINC01811 in the CRC samples and normal samples in the Starbase database. **G** The expression of LINC01811 mRNA in CRC tissues and normal tissues were detected by qRT-PCR assay. ***p < 0.001

To identify lncRNAs exhibiting differential expression between CRC and para-cancerous samples, we conducted a differential expression analysis involving 456 CRC samples and 41 para-cancerous tissues from the TCGA cohort. Compared to the para-cancerous samples, 28 lncRNAs were significantly up-regulated in the CRC samples (Fig. 1A, B). Additionally, we performed differential expression analysis on 41 paired CRC and para-cancerous samples based on lncRNA expression values. In contrast to the para-cancerous samples, 7 lncRNAs were significantly up-regulated and 1 lncRNA was down-regulated in the CRC samples (Fig.  1C, D). Cross-analysis revealed that the expressions of 7 lncRNAs (FEZF1-AS1, AC007099.1, LINC00460, LINC01705, AC025154.2, LINC01811, and AC117386.2) were significantly up-regulated in both the 456 CRC samples (vs. 41 para-cancerous tissues) and the 41 CRC samples (vs. 41 paired para-cancerous tissues) (Fig. 1E). Among these, LINC01811 was significantly up-regulated in CRC samples according to the Starbase database (CRC vs. normal samples) (Fig. 1F). Furthermore, we found that LINC01811 was significantly up-regulated in CRC tissues compared to normal tissues (Fig. 1G, *p*< 0.001). These results suggest that LINC01811 may function as a cancer-promoting factor in CRC.

### LINC01811 silencing inhibited migration and invasion of CRC cells

**Fig. 2:**
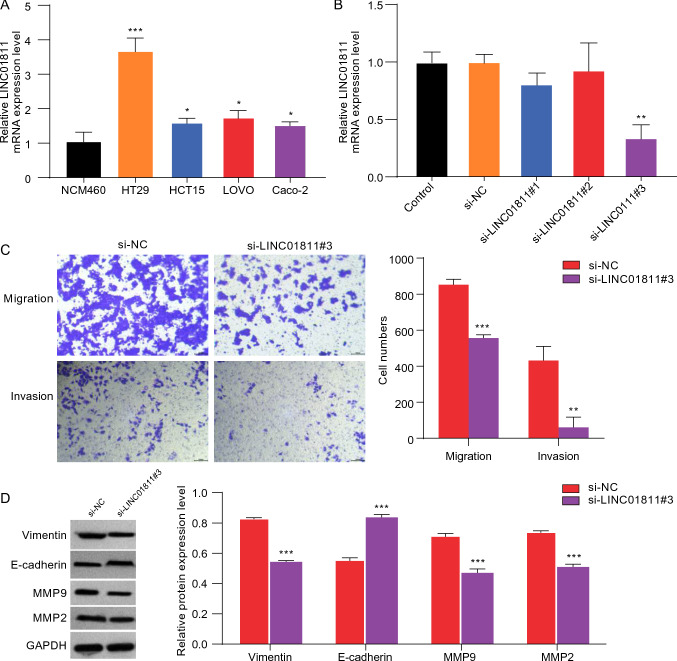
LINC01811 silencing inhibited migration and invasion of CRC cells. **A** The relative mRNA expression level of LINC01811 in cell lines (NCM460, HT29, HCT15, LOVO, Caco-2) was detected using qRT-PCR assay. **B** The knockdown efficiency of LINC01811 was analyzed by qRT-PCR. **C** Migration and invasion of LINC01811 silencing cells were evaluated by Transwell assay. **D** The levels of vimentin, E-cadherin, MMP9, and MMP2 protein expressions were detected by Western blot. *p < 0.05, **p < 0.01, ***p < 0.001

To investigate the biological functions of LINC01811 in the metastasis of CRC, we initially examined the expression of LINC01811 in normal NCM460 cells and various CRC cell lines using RT-qPCR assay. Figure 2A illustrates the significantly elevated levels of LINC01811 in CRC cell lines compared to NCM460 cells. Among these, HT29 exhibited the highest expression of LINC01811 (*p* < 0.001), prompting us to select HT29 cells for subsequent experiments. The role of LINC01811 in promoting metastasis in CRC was assessed by silencing LINC01811 in HT29 cells. The effect of LINC01811 silencing was evaluated using qRT-PCR (Fig. 2B, *p* < 0.01). Additionally, transwell assays demonstrated that the knockdown of LINC01811 led to a significant reduction in the migration and invasion abilities of HT29 cells (Fig. 2C, *p* < 0.05). To further explore the mechanism undering these findings, we examined the impact of LINC01811 on epithelial-mesenchymal transition (EMT) in CRC cells through Western blot analysis. The results indicated that silencing LINC01811 resulted in decreased levels of the mesenchymal marker protein vimentin and increased levels of the epithelial marker protein E-cadherin in HT29 cells (Fig. 2D). Moreover, matrix metalloproteinases (MMPs) are crucial proteolytic enzymes known for their ability to degrade the extracellular matrix (ECM) and have been implicated in promoting tumor progression (Gialeli et al. 2011). MMPs play a significant role in facilitating metastasis and invasion in various cancers, including CRC (Yao et al. 2018). As shown in Fig. 2D, silencing LINC01811 significantly reduced the protein levels of MMP2 and MMP9 in HT29 cells. Collectivelly, these findings suggest that silencing LINC01811 may inhibit the migration and invasion of CRC cells.

### LINC01811 acts as a sponge for miR-214-3p in the CRC cells

**Fig. 3:**
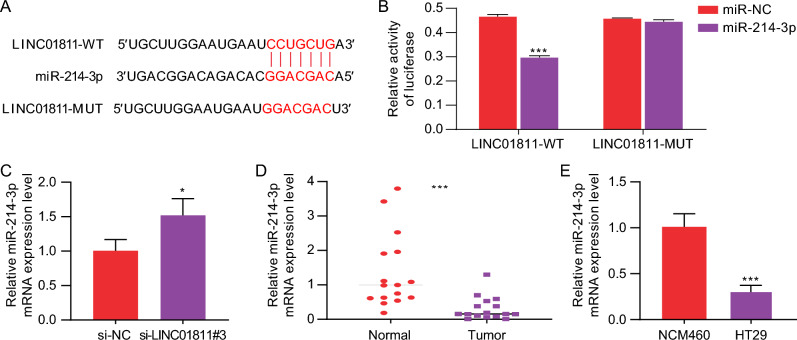
LINC01811 acts as a sponge for miR-214-3p in the CRC cells. **A** The putative binding sites between miR-214-3p and LINC01811 were predicted by starBase. **B** The predicted sites between LINC01811 and miR‐214-3p were identified by dual-luciferase reporter assay. **C** the expression of miR-214-3p in the LINC01811 silencing HT29 cells was detected using qRT-PCR. **D**, **E**. The expression of miR-214-3p in CRC tissues and HT29 cells was detected by RT-qPCR assay. *p < 0.05, ***p < 0.001

LncRNAs act as sponges to modulate gene expression by competitively binding to miRNAs and preventing target miRNA response elements (MREs) from interacting with miRNAs (Zhu et al. 2020). An association between LINC01811 and miR-214-3p was identified in the Starbase database (Fig. 3A). The luciferase reporter assay demonstrated that the overexpression of miR-214-3p significantly reduced the luciferase activity of LINC01811-WT in HT29 cells, while LINC01811-MUT remained unaffected (Fig. 3B, *p* < 0.001). This finding suggests that miR-214-3p may act as a target for LINC01811. In addition, silencing LINC01811 significantly elevated miR-214-3p expression in HT29 cells (Fig. 3C, *p* < 0.05), indicating that LINC01811 negatively regulates miR-214-3p. Furthermore, miR-214-3p expression was found to be decreased in CRC tissues and HT29 cells (Fig. 3D, E,  *p* < 0.001). These findings indicate that miR-214-3p is the target miRNA of LINC01811.

### LINC01811 silencing inhibited the progression of CRC cells by targeting miR-214-3p in vitro

**Fig. 4:**
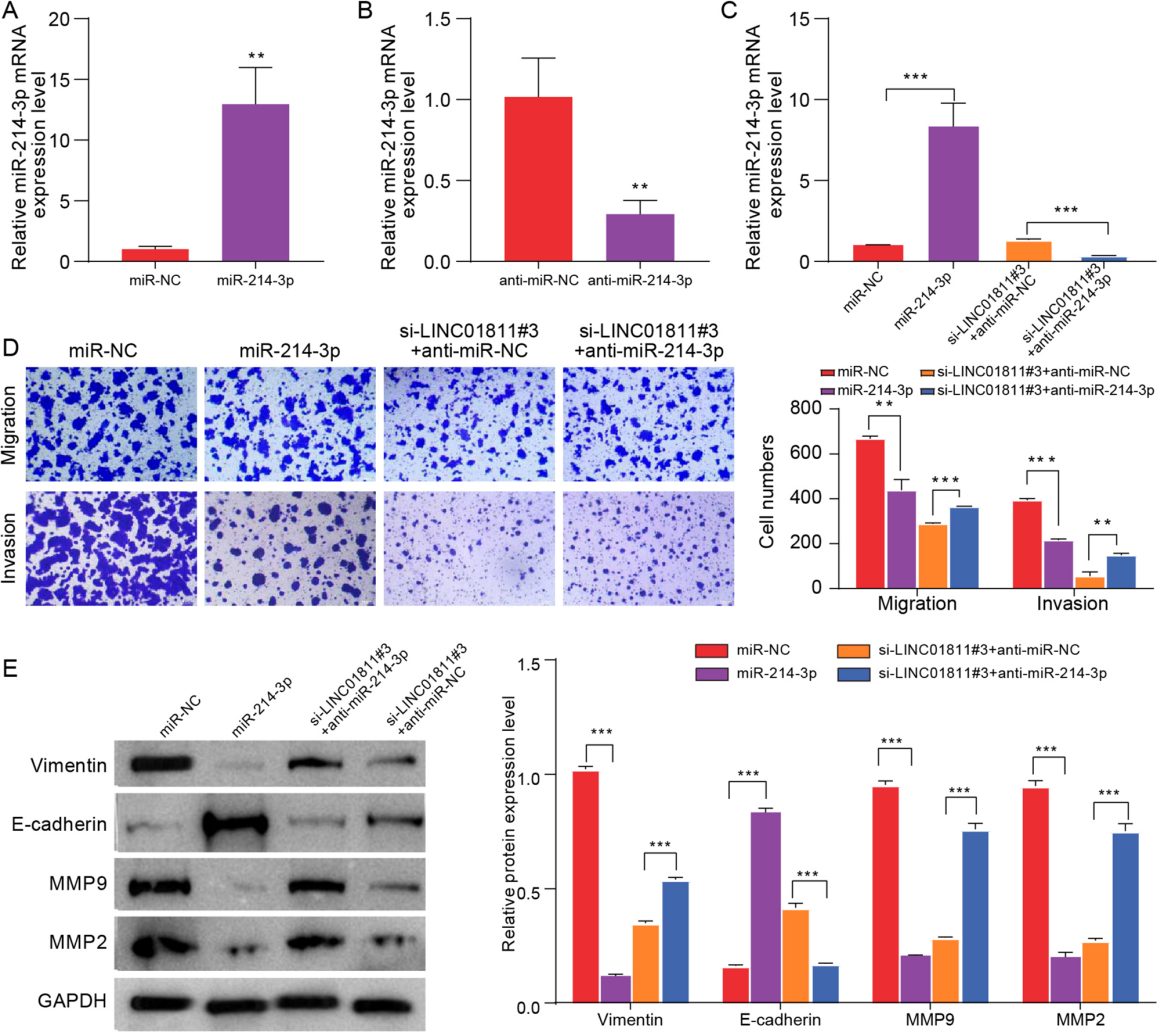
LINC01811 silencing inhibited the progression of CRC cells by targeting miR-214-3p in vitro. **A**, **B**. The overexpression or knockdown efficiency of miR-214-3p in cells was analyzed by qRT-PCR. **C** The expression of miR-214-3p in the different groups was determined by qRT-PCR. **D** Cell migration and invasion were evaluated by transwell assay. **E** The levels of vimentin, E-cadherin, MMP9, and MMP2 protein expressions in each group were detected by Western blot. **p < 0.01, ***p < 0.001

To further explore the relationship between LINC01811 and miR-214-3p, experiments were conducted on HT29 cells involving transfection with miR-NC, miR-214-3p, anti-miR-NC, or anti-miR-214-3p. The results demonstrated a significant increase in miR-214-3p expression in the miR-214-3p group and a decrease in the anti-miR-214-3p groups compared to their respective NC groups (Fig. 4A, B). Subsequent transfection of HT29 cells with miR-NC, miR-214-3p, si-LINC01811#3 + anti-miR-NC, or si-LINC01811#3 + anti-miR-214-3p revealed a decrease in miR-214-3p expression in the si-LINC01811#3 + anti-miR-214-3p group compared to the si-LINC01811#3 + anti-miR-NC group (Fig. 4C). Additionally, it was observed that elevated miR-214-3p levels or LINC01811 silencing effectively inhibited the migration and invasion capabilities of HT29 cells, while the effect of LINC01811 silencing on cell migration and invasion was mitigated by the miR-214-3p inhibitor (Fig. 4D). The overexpression of miR-214-3p or silencing of LINC01811 resulted in a reduction of Vimentin protein expression and an increase in E-cadherin protein expression in HT29 cells, while the impact of LINC01811 silencing was also mitigated by the miR-214-3p inhibitor (Fig. 4E). Moreover, the overexpression of miR-214-3p or silencing of LINC01811 significantly reduced MMP9 and MMP2 protein expression in HT29 cells, while the effect of LINC01811 silencing could be reversed by the miR-214-3p inhibitor (Fig. 4E). These results indicate that LINC01811 silencing may inhibit the progression of CRC cells by targeting miR-214-3p.

### *YAP1* was a target gene of miR-214-3p

**Fig. 5:**
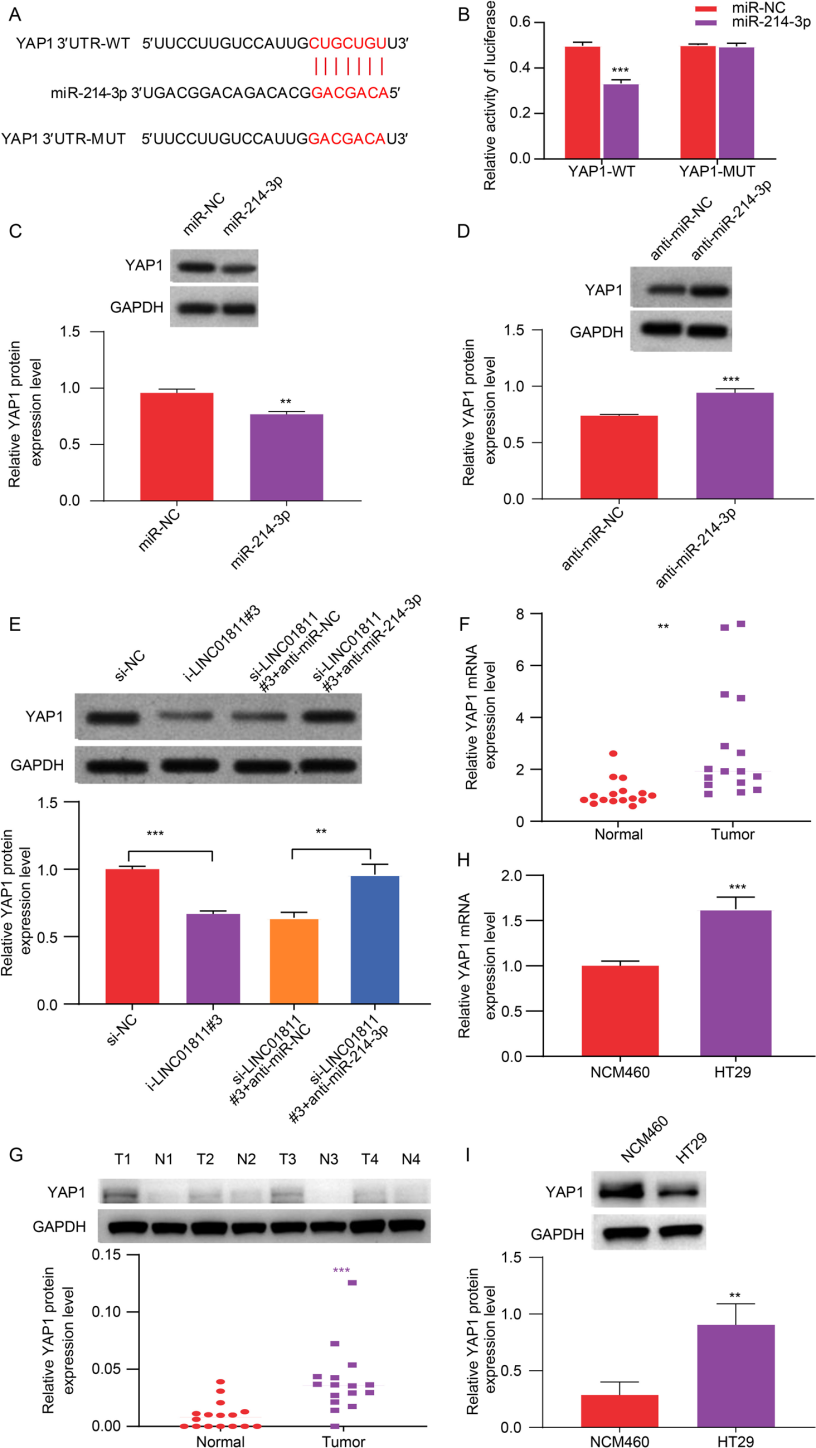
*YAP1* was a target gene of miR-214-3p. **A** The putative binding sites between miR-214-3p and *YAP1* were predicted by starBase. **B** The predicted sites between miR‐214-3p and *YAP1* were identified by dual-luciferase reporter assay. **C**, **D** The expression of *YAP1* in overexpressed miR-214-3p and miR-214-3p inhibitor groups was detected using Western blot. **E** The expression of *YAP1* protein in each group was detected by Western blot. **F**–**H** The expression of *YAP1* mRNA in CRC tissues and cell lines was determined by qRT-PCR. **G**–**I** The expression of YAP1 protein in CRC tissues and cell lines was detected using Western blot.**p < 0.01, ***p < 0.001

In the starBase database, *YAP1* was identified as a potential target of miR-214-3p (Fig. 5A). Subsequently, miR-214-3p mimics and inhibitors were co-transfected with *YAP1*-WT or *YAP1*-MUT into HT29 cells, followed by a dual luciferase reporter assay. The results demonstrated that the overexpression of miR-214-3p effectively decreased the luciferase activity in YAP1-WT cells, with no impact obaerved in *YAP1*-MUT cells (Fig. 5B). In HT29 cells, the overexpression of miR-214-3p led to a reduction in YAP1 expression, while inhibition of miR-214-3p resulted in an increase in *YAP1* expression (Fig. 5C, D). Moreover, silencing of LINC01811 significantly decreased *YAP1* expression, a change that could be reversed by the miR-214-3p inhibitor (Fig. 5E). Examination of YAP1 mRNA and protein expression levels in CRC tissues and HT29 cells revealed an increase in YAP1 expression (Fig. 5F–I). These findings further support the potential regulatory role of miR-214-3p in modulating *YAP1* expression in CRC.

### Overexpressed *YAP1* reversed the effects of LINC01811 silencing on CRC cells

**Fig. 6:**
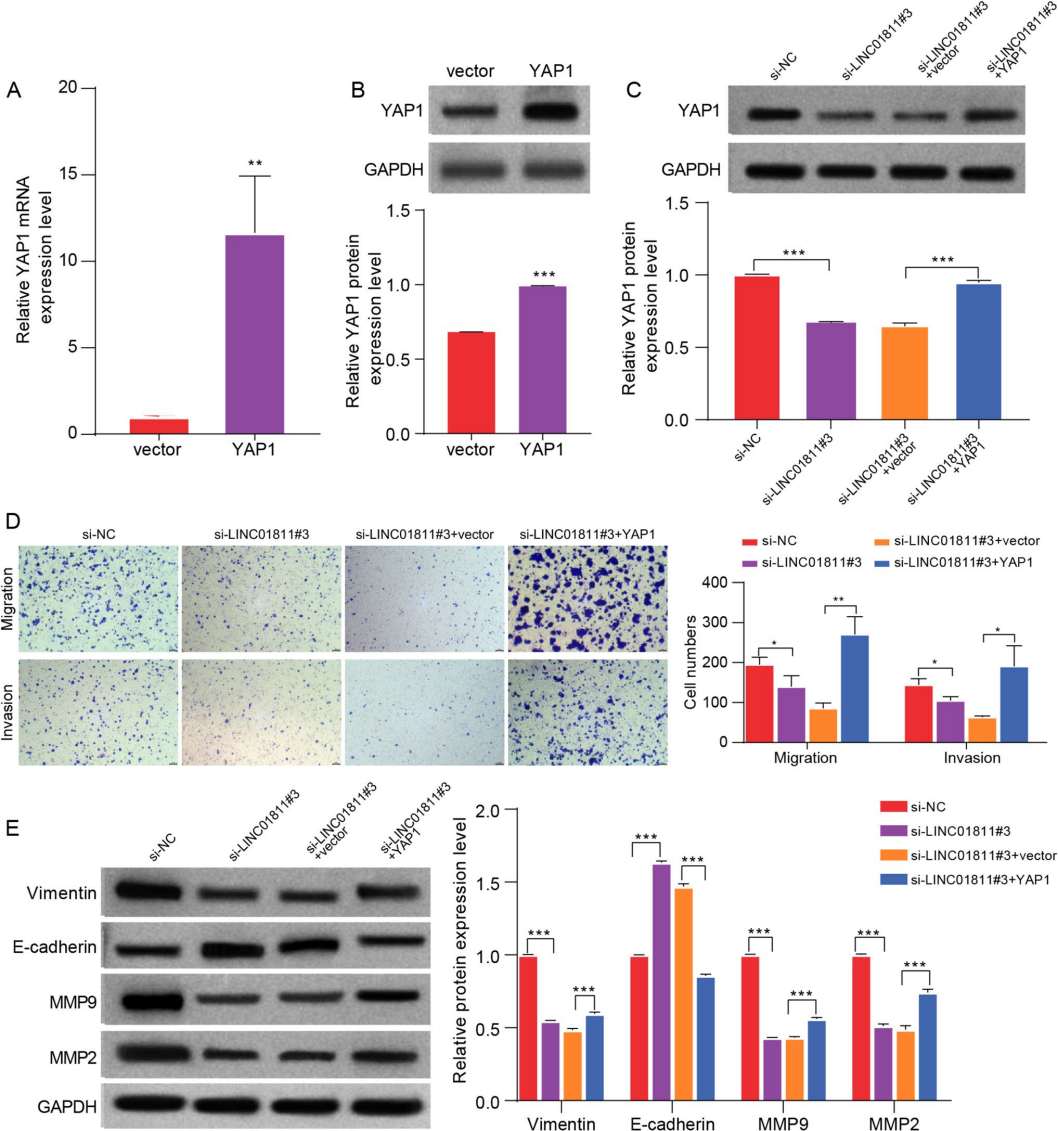
Overexpressed *YAP1* reversed the effects of LINC01811 silencing on CRC cells. **A**, **B** The overexpression efficiency of YAP1 mRNA and protein in HT29 dells was analyzed by qRT-PCR and Western blot, respectively. **C** The expression of YAP1 protein in each group was detected using Western blot. **D** Cell migration and invasion in each group were evaluated by transwell assay. **E** The levels of vimentin, **E** The expression level of cadherin, MMP9, and MMP2 protein in each group were detected by Western blot. *p < 0.05, **p < 0.01, ***p < 0.001

To investigate the functional relationship between LINC01811 and *YAP1*, we first established a cell model with overexpressed *YAP1*. As shown in Fig. 6A, B, compared with the vector control group, the OE-*YAP1* group exhibited a significant increase in both *YAP1* mRNA and protein levels. Subsequently, HT29 cells were transfected with si-NC, si-LINC01811#3, si-LINC01811#3 + vector, or si-LINC01811#3 + OE-*YAP1*, respectively. The results revealed that silencing of LINC01811 markedly reduced *YAP1* protein expression. However, this inhibitory effect was abolished by overexpression of *YAP1* (Fig. 6C). Furthermore, the inhibitory effects of LINC01811 silencing on cell migration and invasion were reversed by *YAP1* overexpression (Fig. 6D). Additionally, the suppressive effects of LINC01811 silencing on the expression of vimentin, MMP9, and MMP2, as well as the promoting effect on E-cadherin expression, were all abolished by *YAP1* overexpression (Fig. 6E). Collectively, these findings demonstrate that overexpressed *YAP1* can counteract the effects of LINC01811 silencing on CRC cells in vitro.

## Discussion

Several studies have highlighted the significance of lncRNAs in the context of tumor-related biomolecules and their functional roles in CRC progression. For instance, Zhu et al. observed overexpression of lncRNAs RP11-757G1.5 and RP11-51O6.1 in CRC tissues, with higher levels of these lncRNAs being correlated with worse clinical outcomes in CRC patients (Zhu et al. 2020, 2021). Furthermore, the increased expression of RP11-757G1.5 or RP11-51O6.1 was associated with enhanced proliferation of CRC cells, indicating their potential role in cancer advancement (Zhu et al. 2020, 2021). In addition, LINC02418 was identified as being significantly upregulated in chemoresistant CRC cells, and targeted silencing of this lncRNA resulted in improved sensitivity to chemotherapy in HCT8/5-Fu and HCT8/DDP cells (Yao et al. 2022), indicating LINC02418 might play a role in mediating resistance to chemotherapy in CRC. In addition, a study by Liu et al. demonstrated that EIF3J-AS1 expression was increased in CRC samples, indicating a negative outcome for CRC patients (Liu et al. 2020). Suppression of EIF3J-AS1 had a notable impact on decreasing CRC cell proliferation and enhancing apoptotic activity (Liu et al. 2020). Therefore, exploring additional crucial lncRNAs involved in CRC development is a valuable pursuit.

To identify potential functional lncRNAs, we analyzed publicly available microarray data associated with CRC. Our analysis revealed a novel lncRNA, LINC01811, that exhibited significantly higher expression levels in CRC tissues and cells. Currently, there are only a few reports about the mechanism of LINC01811 in the development of cancers. Li et al. reported that LINC01811 had a strong diagnostic value for epithelial ovarian cancer (Li and Li 2022). This study found that LINC01811 silencing markedly reduced the migration and invasion in HT29 cells. EMT is a biological process where epithelial cells undergo specific procedures to transform into mesenchymal phenotypes. This process can result in the loss of epithelial cell polarity, decreased adhesion between tumor cells, and enhanced migration of tumor cells away from the primary site, ultimately facilitating tumor invasion and metastasis (Brabletz et al. 2018). We found that the reduction of LINC01811 expression resulted in a notable decrease in the expression of the mesenchymal marker protein vimentin and a subsequent increase in the expression of the epithelial marker protein E-cadherin within CRC cells. Additionally, the ECM plays a crucial role in impeding the invasion and metastasis of tumor cells by affecting their mobility (Walker et al. 2018). Excessive ECM concentration can lead to a significant increase in adhesion force, hindering the cell’s ability to migrate further (Walker Mojares et al. 2018). MMPs are primary proteolytic enzymes responsible for degrading the ECM and have been demonstrated to be crucial in facilitating the migration and spread of cancer cells (Bonnans et al. 2014). MMPs could activate TGF-β and promote EMT (Gialeli et al. 2011). In our study, we discovered that LINC01811 silencing remarkably reduced MMP2 and MMP9 protein levels in CRC cells. Overall, these results indicated that silencing ANKHD1 might reduce CRC cell migration and invasion by inhibiting EMT.

lncRNAs can act as competing endogenous RNAs (ceRNAs) by binding to miRNAs and affecting their ability to regulate gene expression (Zhu et al. 2020). For example, SEAS1 was shown to regulate cancer progression in triple-negative breast cancer by modulating miR-3940-3p levels (Hu et al. 2022). Similarly, in triple-negative breast cancer, the SEMA3B-AS1 plays a role as a decoy for miR-3940-3p to inhibit the degradation of its target gene KLLN (Hu et al. 2022). Our study revealed that LINC01811 targeted miR-214-3p and negatively regulated its expression. Previous research has highlighted the significance of miR-214-3p in CRC progression. Zhou and colleagues showed that lower miR-214-3p levels in CRC samples were linked to increased tumor size and the spread of cancer cells to nearby lymph nodes (Zhou et al. 2020). Knockdown of miR-214-3p was found to enhance cell growth and metastasis in CRC (Zhou et al. 2020), while overexpression of miR-214-3p inhibited cell proliferation, migration, and invasion (Ni et al. 2019a, b). We also found that overexpressed miR-214-3p remarkably reduced CRC cell migration and invasion. Moreover, our current study found that the miR-214-3p expression was low in CRC samples, and similar results of miR-214-3p in CRC were discovered in the previous studies (Zhou, Wu et al., 2020). Function assays demonstrated that silencing LINC01811 inhibited cell migration and invasion, downregulated the expression of Vimentin, MMP9, and MMP2 proteins, and upregulated the expression of E-cadherin protein. Furthermore, the inhibitory effect of LINC01811 silencing was reversed by a miR-214-3p inhibitor. The results above suggested that LINC01811 might had a crucial impact on invasion and migration in CRC advancement by acting as a sponge for miR-214-3p in vitro.

Several studies have indicated that miRNA plays a crucial role in promoting CRC carcinogenesis by modulating the *YAP1* signaling pathway. For example, the RP11-757G1.5 gene modulated *YAP1* expression by interacting with miR-139-5p, thereby enhancing the proliferation and metastasis of CRC cells (Zhu, Bu et al., 2020). Similarly, EIF3J-AS1 upregulated *YAP1* levels by sequestering miR-3163, leading to increased proliferation and reduced apoptosis in CRC cells (Liu et al. 2020). Our research revealed a high expression of *YAP1* in both CRC tissues and cells, corroborating previous findings (Ou et al. 2020). Interestingly, miR-214-3p was identified as a regulator of *YAP1*. Silencing LINC01811 significantly reduced *YAP1* expression, which was rescued by an inhibitor of miR-214-3p. Functional assays demonstrated that *YAP1* overexpression mitigated the effects of LINC01811 silencing on cell migration and invasion. Furthermore, upregulating *YAP1* reversed the impact of LINC01811 silencing on Vimentin, MMP9, and MMP2 levels, while increasing E-cadherin expression. These findings suggested that LINC01811 likely influences CRC progression by targeting the miR-214-3p/*YAP1* pathway.

## Conclusion

In conclusion, our study identified a new lncRNA, LINC01811, which showed significant up-regulation in CRC tissues and cells. Inhibition of LINC01811 led to decreased migration and invasion of CRC cells. Furthermore, our research revealed that LINC01811 functions as an oncogene by acting as a sponge for miR-214-3p, resulting in increased expression of YAP1. Overall, our findings highlight the potential of LINC01811 as a promising therapeutic target for CRC.

## Supplementary Information

Below is the link to the electronic supplementary material.

Supplementary file1 (DOCX 15 KB)

## Data Availability

Publicly available datasets used in this study can be found in The Cancer Genome Atlas (TCGA, https://tcga-data.nci.nih.gov/tcga/) database. The experimental data supporting the results of this study are available from the corresponding author upon reasonable request.
