# Supplementary material for: Long noncoding RNA LINC01811 sponges miR-214-3p and upregulates YAP1 thereby promoting the migration and invasion of colorectal cancer
Source: 3 Biotech. 2025 Apr 10;15(5):123. doi: 10.1007/s13205-025-04292-8 (PMC11985869; doi:10.1007/s13205-025-04292-8)
Supplement: Supplementary file 1 — Supplementary file1 (DOCX 15 KB) [file 13205_2025_4292_MOESM1_ESM.docx]

**Table S1** The clinical information of patients

| **No.** | **Age** | **Gender** | **Tumor Size (cm×cm)** | **T** | **N** | **M** | **Differentiation** |
| --- | --- | --- | --- | --- | --- | --- | --- |
| 1 | 59 | Male | 5.5cm | 3 | 0 | 0 | Moderately |
| 2 | 54 | Male | 6cm | 3 | 1 | 0 | Moderately |
| 3 | 55 | Male | 2.7cm | 2 | 0 | 0 | Moderately |
| 4 | 68 | Male | 3.4cm | 3 | 2 | 0 | Moderately |
| 5 | 52 | Female | 4cm | 3 | 2 | 0 | Moderately |
| 6 | 77 | Female | 3.5cm | 3 | 2 | 0 | Moderately |
| 7 | 80 | Male | 2.3cm | 1 | 1 | 0 | Moderately |
| 8 | 72 | Female | 2.5cm | 3 | 1 | 0 | Moderately |
| 9 | 67 | Female | 6.5cm | 3 | 0 | 0 | Moderately |
| 10 | 73 | Female | 4.5cm | 2 | 0 | 0 | Moderately |
| 11 | 70 | Male | 4.8cm | 3 | 0 | 0 | High |
| 12 | 79 | Male | 4cm | 3 | 2 | 0 | Moderately |
| 13 | 51 | Female | 3.3cm | 2 | 0 | 0 | Low |
| 14 | 64 | Male | 3.6cm | 2 | 1 | 0 | Moderately |
| 15 | 59 | Male | 6.0cm | 3 | 1 | 0 | Low |
